# BMC *Neuroscience* reviewer acknowledgement 2014

**DOI:** 10.1186/s12868-015-0139-5

**Published:** 2015-02-17

**Authors:** Tim Shipley

**Affiliations:** BioMed Central, Floor 6, 236 Gray’s Inn Road, London, WC1X 8HB UK

**Keywords:** ■■■

## Abstract

The editors of BMC Neuroscience would like to thank all our reviewers who have contributed to the journal in Volume 15 (2014).

**Paula Agostinho**

Portugal

**Saif Ahmad Saudi**

Arabia

**Ludwig Aigner**

Austria

**Michael Akins**

United States of America

**Hakan Aldskogius**

Sweden

**John Allen**

United States of America

**Silvana Allodi**

Brazil

**Julià L Amengual**

Spain

**Miroslava Anderova**

Czech Republic

**Balasubramaniam Annamalai**

United States of America

**Saema Ansar**

Sweden

**Ubaldo Armato**

Italy

**Mariana Astiz**

Spain

**Ilknur Ay**

United States of America

**Travis Baker**

Canada

**Veera Venkata Ratnam Bandaru**

United States of America

**Rina Bandopadhyay**

United Kingdom

**Andrew Bankston**

United States of America

**Jianxin Bao**

United States of America

**Guido Barchiesi**

Italy

**Anirban Basu**

India

**Nadhim Bayatti**

United Kingdom

**Richard Bazinet**

United States of America

**M. Flint Beal**

United States of America

**Maik Behrens**

Germany

**Christian Bellebaum**

Germany

**Gian Carlo Bellenchi**

Italy

**Kerstin Bendfeldt**

Switzerland

**Itai Berger**

Israel

**Sablonniere Bernard**

France

**Neda Bernasconi**

Canada

**Hans-Gert Bernstein**

Germany

**John Bethea**

United States of America

**Pallab Bhattacharya**

India

**Knut Biber**

Germany

**Patrick Blader**

France

**Nico Boehler**

United States of America

**Corina Bondi**

United States of America

**Cesar Borlongan**

United States of America

**Marco Bortolato**

United States of America

**Yvonne Böttcher**

Germany

**Matthew Boyko**

Israel

**Patrick Bradshaw**

United States of America

**Clive Bramham**

Norway

**Roland Brandt**

Germany

**Filippo Brighina**

Italy

**Guy Brock**

United States of America

**Brad Broughton**

Australia

**Emmanuel Brouillet**

France

**Russell Brown**

United States of America

**Anna Bukiya**

United States of America

**Nico Bunzeck**

Germany

**Malgorzata Burek**

Germany

**Josh Burk**

United States of America

**Nikita Burke**

Ireland

**Kimberly Byrnes**

United States of America

**Laura Calza**

Italy

**Joshua Cameron**

United States of America

**Antoni Camins**

Spain

**Simona Capsoni**

Italy

**Filippo Caraci**

Italy

**Marina Cardano**

Italy

**Thierry Charlier**

United States of America

**Aiqing Chen**

United Kingdom

**Huafu Chen**

China

**Sidi Chen**

United States of America

**Sk Cheong**

Malaysia

**Glyn Chidlow**

Australia

**Shinung Ching**

United States of America

**Andrew Chisholm**

United States of America

**Byung Tae Choi**

Korea, South

**Winyoo Chowanadisai**

United States of America

**Tumul Chowdhury**

Canada

**Sylvie Claeysen**

France

**Marc Claret**

Spain

**David Clayton**

United Kingdom

**Luciano Conti**

Italy

**Richard Coppola**

United States of America

**Adam Paul Croft**

United Kingdom

**Li Dai**

United States of America

**Radhouane Dallel**

France

**Andrew Davies**

United Kingdom

**Max De Leeuw**

Netherlands

**Peter De Lissa**

Australia

**Amanda De Oliveira**

Brazil

**Cecile Delarasse**

France

**Cinzia Dello Russo**

Italy

**Catherine Dermon**

Greece

**Stéphanie Deroo**

Belgium

**Paula Desplats**

United States of America

**Megan Detloff**

United States of America

**Tajinder Dhammu**

United States of America

**Narender Dhingra**

India

**James Dillman**

United States of America

**Dragan Djuric**

Serbia

**Robert Donati**

United States of America

**Sarah Donohue**

United States of America

**Ayse Dosemeci**

United States of America

**Rose Du**

United States of America

**Dan Dumitrascu**

Romania

**Valentina Echeverria**

United States of America

**James Christopher Edgar**

United States of America

**Ruben Eggers**

Netherlands

**Andrew Elliot**

United States of America

**James Eubanks**

Canada

**Jim Fadel**

United States of America

**Antonio Faiella**

Italy

**Tracy Farr**

Germany

**Mauro Fasano**

Italy

**S Hossein Fatemi**

United States of America

**Francisco Jose Fernandez Gomez**

France

**Isidro Ferrer**

Spain

**Caleb Finch**

United States of America

**Esther Florin**

Germany

**Steven Frucht**

United States of America

**Bozena Gabryel**

Poland

**Ricardo Galhardoni**

Brazil

**Frederick Gallun**

United States of America

**Olga Lucía Gamboa Arana**

Germany

**Leontien Geven**

Netherlands

**Othman Ghribi**

United States of America

**Alain Ghysen**

France

**Rashid Giniatullin**

Finland

**De Sarro Giovambattista**

Italy

**Massimo Girelli**

Italy

**Anna Göbel**

Germany

**Robert Göder**

Germany

**Martin Goettlich**

Germany

**Giorgio Gorini**

United States of America

**Laura Grabel**

United States of America

**Jessica Green**

United States of America

**Joel Greenberg**

United States of America

**Elke Griesmaier**

Austria

**David Hampson**

Canada

**Jörg Hanrieder**

Sweden

**Jakob Hansen**

Denmark

**Thalía Harmony**

Mexico

**Joseph Harris**

Germany

**Gesa Hartwigsen**

Germany

**Steffen Harzsch**

Germany

**Satoru Hayasaka**

United States of America

**Shuhan He**

United States of America

**Ying He**

United States of America

**Geoff Head**

Australia

**Terry Hebert**

Canada

**Mike Heilemann**

Germany

**Marcus Heldmann**

Germany

**Stefan Heller**

United States of America

**Niels Hellings**

Belgium

**Istvan Hernadi**

Hungary

**Johannes Hewig**

Germany

**Chindo Hicks**

United States of America

**Jens Hjerling Leffler**

Sweden

**Jihane Homman-Ludiye**

Australia

**Dewen Hu**

China

**Hsien-Sung Huang**

United States of America

**Rene Hurlemann**

Germany

**Miki Igarashi**

United States of America

**Yuzuru Ikeda**

Japan

**Joji Inamasu**

Japan

**Yasuhiro Ishihara**

Japan

**Masako Isokawa**

United States of America

**Etsuro Ito**

Japan

**Michael Jackson**

Canada

**Norbert Jausovec**

Slovenia

**Suresh Jesuthasan**

Singapore

**Meiyan Jiang**

United States of America

**Dirk Junghans**

Switzerland

**Mohamed Kabbaj**

United States of America

**John Kalbfleisch**

United States of America

**Takashi Kanda**

Japan

**Sang Soo Kang**

Korea, South

**Ilia Karatsoreos**

United States of America

**Charanjit Kaur**

Singapore

**Amajad Kazi**

India

**Mohammad Moshahid Khan**

United States of America

**Rajesh Khanna**

United States of America

**June Sic Kim**

Korea, South

**Jieun Kim**

United States of America

**Yosuke Kita**

Japan

**Katrin Klebermaß-Schrehof**

Austria

**Anthony Kline**

United States of America

**Ulrike Krämer**

Germany

**Stefanie Kuerten**

Germany

**Yu-Min Kuo**

Taiwan

**Charalambos Kyriacou**

United Kingdom

**Madepalli Lakshmana**

United States of America

**Thomas Lanz**

United States of America

**Robert Ledeen**

United States of America

**Soon-Tae Lee**

United States of America

**Patrick Lewis**

United Kingdom

**Bingcang Li**

China

**Yonggang Li**

United States of America

**Stefan Liebau**

Germany

**Monika Liguz-Lecznar**

Poland

**Weihong Lin**

United States of America

**Eng-Ang Ling**

Singapore

**Jonathan List**

Germany

**Hao Liu**

United States of America

**Guangming Lu**

China

**Fang Luo**

China

**Wenbo Luo**

China

**Wenyu Luo**

United States of America

**Svetlana Lutsenko**

United States of America

**Marzena Mackowiak**

Poland

**Mark Majchrzak**

United States of America

**David Male**

United Kingdom

**Volker Mall**

Germany

**Ml Mansego**

Spain

**Serge Marbacher**

Switzerland

**Paula Martin**

United States of America

**Mathew Martin-Iverson**

Australia

**Hirofumi Maruyama**

Japan

**Tessa Marzi**

Italy

**Eliezer Masliah**

United States of America

**Colin Masters**

Australia

**Fumiyo Matsuda**

Japan

**Gianluca Matteoli**

Belgium

**Gianluigi Mazzoccoli**

Italy

**Laura Medina-Ceja**

Mexico

**Suresh Mehta**

United States of America

**Dies Meijer**

Netherlands

**Onno Meijer**

Netherlands

**Noam Meiri**

Israel

**Mareike Menz**

Germany

**Francisco Mercado**

Spain

**David Miller**

United States of America

**Kenneth Miller**

United States of America

**Richard Milner**

United States of America

**Teresa Mitchell**

United States of America

**Seiji Miyata**

Japan

**Sumiko Mochida**

Japan

**Matthias Mölle**

Germany

**Julio Cesar Morales Medina**

Mexico

**Gabriela Morali**

Mexico

**Joe Moran**

United States of America

**Kyoji Morita**

Japan

**Marija Mostarica-Stojkovic**

Serbia

**Bernadette Murphy**

Canada

**Shin Nagayama**

United States of America

**Kazuki Nagayasu**

Japan

**Pradeep Naik**

India

**Raad Nashmi**

Canada

**Xavier Navarro**

Spain

**Igor Nenadic**

Germany

**Winfried Neuhaus**

Austria

**Guilherme Neves**

United Kingdom

**Vadim Nikulin**

Germany

**Johannes Nimpf**

Austria

**Sharon Oneill**

United States of America

**Wei-Yi Ong**

Singapore

**Marco Onorati**

United States of America

**Jullie Pan**

United States of America

**Vinay Parikh**

United States of America

**Surojit Paul**

United States of America

**Rosetta Pedotti**

Italy

**Louis Penning**

Netherlands

**Isabel Perez Otaño**

Spain

**Stefanie Peykarjou**

Germany

**Dzung Pham**

United States of America

**Linda Phillips**

United States of America

**Emmanuel Pinteaux**

United Kingdom

**Paulo Pires**

United States of America

**Justin Piro**

United States of America

**Cristina Porras**

Spain

**Christine Portfors**

United States of America

**Shuhong Qiao**

United States of America

**Guoping Qing**

China

**Mario Quarantelli**

Italy

**Nidia Quillinan**

United States of America

**Monika Rabenstein**

Germany

**Jacob Raber**

United States of America

**Peter Racay**

Slovakia

**Biserka Radosevic-Stasic**

Croatia

**Valeria Ramaglia**

Netherlands

**Murali Ramanathan**

United States of America

**Cedric Raoul**

France

**P. Hemachandra Reddy**

United States of America

**Eva Redei**

United States of America

**George Reeke**

United States of America

**Lina Reiss**

United States of America

**Renate Reniers**

United Kingdom

**Mathias Rhein**

Germany

**Alfredo Ribeiro-Da-Silva**

Canada

**Jeremy Rich**

United States of America

**William Richardson**

United Kingdom

**Ulrike Rimmele**

Switzerland

**Maja Rogic**

Croatia

**Patricia Rojas**

Mexico

**Mikael Roll**

Sweden

**Alejandro Romero**

Spain

**Aribert Rothenberger**

Germany

**Eugeni Roura**

Australia

**Ruth Ruscheweyh**

Germany

**Gesine Saher**

Germany

**Manabu Sakakibara**

Japan

**Harutoshi Sakakima**

Japan

**Dan Sanes**

United States of America

**Kazunori Sango**

Japan

**Kazunobu Sawamoto**

Japan

**Mohammad Sayyah**

Iran

**Mya Schiess**

United States of America

**Felix Schlachetzki**

Germany

**Matthias Schlesewsky**

Germany

**Alexander Schmidt**

Germany

**Leonid Schneider**

Germany

**Mircea Ariel Schoenfeld**

Germany

**Stefanie Schreiber**

Germany

**Markus Schubert**

Germany

**Enrico Schulz**

United Kingdom

**Sarah Schuster**

Austria

**Karen Scott**

Ireland

**Tommy Seaborn**

Canada

**Nicolas Sergeant**

France

**Bridget Shafit-Zagardo**

United States of America

**Zahoor Shah**

United States of America

**Rebecca Shansky**

United States of America

**Anandakumar Shunmugavel**

United States of America

**Bai Chuang Shyu**

Taiwan

**James Sikela**

United States of America

**Luigi Sironi**

Italy

**David Slattery**

Germany

**Robert Smith**

United States of America

**Po-Wah So**

United Kingdom

**Wesley Solomon**

United States of America

**Enrique Soto**

Mexico

**Kim Staats**

Belgium

**Mark Stahl**

United States of America

**Jeannette Stankowski**

United States of America

**Axel Steiger**

Germany

**Ivana Stevanovic**

Serbia

**Jerry Stitzel**

United States of America

**Judith Strong**

United States of America

**Aideen Sullivan**

Ireland

**Joanna Sypecka**

Poland

**Gabor Szabo**

Hungary

**Changiz Taghibiglou**

Canada

**Saeid Taheri**

United States of America

**Nobuyuki Takei**

Japan

**Andrew Tapper**

United States of America

**Silke Telkemeyer**

Germany

**Charlotte Teunissen**

Netherlands

**Wipawan Thangnipon**

Thailand

**Ronald Thomas**

United States of America

**Christopher Thompson**

United States of America

**Veronika Thorns**

Germany

**Lixia Tian**

China

**Xuebi Tian**

China

**Piotr Topilko**

France

**Germán Torregrosa**

Spain

**Pablo Torterolo**

Uruguay

**Joseph Tracy**

United States of America

**Richa Tripathi**

United Kingdom

**Vladimir Tsibulsky**

United States of America

**Oliver Tuescher**

Germany

**Kwong-Chung Tung**

Taiwan

**Bradley Turner**

Australia

**Ann Turnley**

Australia

**Henning Ulrich**

Brazil

**Elias Utreras**

Chile

**Fred Van Leuven**

Belgium

**Marie-José Van Tol**

Netherlands

**Alessandro Vercelli**

Italy

**Marjolein Verly**

Belgium

**Matthijs Vink**

Netherlands

**Logan Voss New**

Zealand

**Pascal Vrticka**

United States of America

**Esther Walton**

Germany

**Chao Wang**

United States of America

**Chengzhong Wang**

United States of America

**Dan Wang**

Japan

**Gang Wang**

United States of America

**Tongfei Wang**

United States of America

**Xiaoxi Wang**

United States of America

**Tracy Warbrick**

Germany

**Katrin Willig**

Germany

**David Wirtshafter**

United States Minor Outlying Islands

**Yung Hou Wong**

China

**Kin Foon Kevin Wong**

United States of America

**Eligiusz Wronka**

Poland

**Ching Hsiang Wu**

Taiwan

**Anhua Wu**

China

**Xu Xue-Bing**

China

**Ipek Yalcin Christmann**

France

**Kenji Yamamoto**

Japan

**Yongjie Yang**

United States of America

**Yongquan Ye**

United States of America

**Shan Ping Yu**

United States of America

**Zengqiang Yuan**

China

**Ariane Zamoner**

Brazil

**Xia Zhang**

Canada

**Yumin Zhang**

United States of America

**Deming Zhao**

China

**Hongling Zhu**

United States of America

**Lei Zhu**

United States of America

**Ling-Qiang Zhu**

China

**Xi-Nian Zuo**

China

